# "To Choose a Band-Aid" - Children’s Preferences for Participation in Health Care Situations: Q Methodology Study

**DOI:** 10.2196/89802

**Published:** 2026-04-21

**Authors:** Merja Hietanen, Maksims Kornevs, Catarina Nahlén Bose, Jayanth Raghothama, Sebastiaan Meijer

**Affiliations:** 1 Swedish Red Cross University, Hälsovägen 11 C, Huddinge, Stockholm, 141 57, Sweden, 46 08-587 516 60; 2 KTH Royal Institute of Technology, Huddinge, Stockholm, Sweden

**Keywords:** children, child participation, child's perspective, health care, Q methodology

## Abstract

**Background:**

Child-centered care (CCC) is standard practice in pediatrics, emphasizing the child as an individual with rights while acknowledging the child’s role within the family. A key aspect of CCC is the involvement of the child in health care decisions alongside parents and professionals. Although this is a right recognized by the United Nations Convention on the Rights of the Child, it may not always be applied in practice.

**Objective:**

The aim of this study is to explore the participation preferences of 3- to 5-year-old children in health care from both their own viewpoint (the child’s perspective) and the viewpoint of their parents and health care professionals.

**Methods:**

Q methodology was used to study preferences, comparing responses from 12 children, 14 parents, and 12 health professionals who ranked 25 statements about ways in which children could participate in health care situations. Factor analysis was used to reveal similarities and differences in views on participation preferences. The children’s rankings were also analyzed separately for comparison.

**Results:**

Analysis of rankings from children, their parents, and health care professionals identified 3 perspectives with different preferences: direct communication between the child and healthcare professionals; understanding and shared decision-making; and responsive and child-led participation. A separate analysis of children’s rankings resulted in 3 perspectives: being included in and setting their own terms for participation; small choices, meaningful outcomes; and trust through familiarity and shared decision-making.

**Conclusions:**

This study suggests that children value shared decision-making and situational control but prefer to leave major decisions to adults. It affirms that preschool-aged children can meaningfully participate in health care when given age-appropriate choices, support, and tools. Children’s perspectives must be acknowledged directly rather than adults assuming their views. The findings support CCC principles and reinforce the United Nations Convention on the Rights of the Child mandate to respect children’s views regarding all issues relevant to them.

## Introduction

Child-centered care (CCC) is the standard focus of care in pediatrics [1]. This concept recognizes the individual child at the center of care as a social agent with rights, but also as part of a family [1,2]. The involvement of children in their health care concerns and decisions alongside parents and health care professionals (HCPs) is central to CCC [2]. Child participation can be viewed from 2 different perspectives: the child’s perspective and the child perspective [3], which are succinctly different in the person formulating the perspective. The child’s perspective refers to a subjective view of a child’s understanding of the world through their perceptions and experiences [4]. The child perspective looks at the same situation through the adult’s perspective by trying to find the child’s point of view, but it is still an adult’s interpretation of the child [4]. Both perspectives must be considered when implementing CCC [5,6], including the adult’s view of the child’s best interest and the child’s own preferences for the situation [6].

Despite its common usage, the term “participation” has different meanings in different academic and professional disciplines and settings. It can refer to anything from physical presence to activity, competence, and a sense of self [7]. Participation can also mean children’s ability to form and express their opinions and influence matters that affect them [8], which is in line with Article 12 of the United Nations Convention on the Rights of the Child (UNCRC) [9]. While the declaration does not specify an age limit for children’s participation, it does state that their age and maturity should be taken into account. Their opinions should be taken seriously and not dismissed due to their age [9]. In this study, the participation of preschool- and school-aged children in health care situations is defined as deciding how to act, receiving adequate information, making decisions, as well as communicating with HCPs [10].

Previous research shows that children are competent agents who can construct their social worlds in collaboration with others [11,12]. They want to be involved in decision-making processes [13,14], yet they are often unable to participate or express their views [11,15,16]. Parents and HCPs play an important role in facilitating children’s participation [10]. However, research has found that parents and HCPs limit children’s opportunities to participate in health care [15,17,18], their opinions are not asked for [15], there is no agreement on the level of children’s participation [11], or parents protect their children from decision-making instead of allowing shared decision-making [19]. A recent review revealed a knowledge gap concerning preschool-aged children’s preferences for participation in health care situations [10]. In accordance with their rights as stated in the UNCRC [9], more research should be conducted into children’s perspectives and participation in health care situations, since this area has not been sufficiently explored. Therefore, it is important to better understand how children prefer to participate in health care situations.

The Swedish National Child Health Care (CHC) program provides free, voluntary health care for children from birth to 6 years of age [20]. This study was conducted in Child Welfare Centers, which are part of the program. It explores the ways in which children prefer to participate in health care situations, focusing on preschool-aged children (3‐5 years old). It examines the topic from 2 perspectives: the child’s perspective, that is, the views of the children themselves, and from the child perspective, that is, the views of their parents and HCPs. It explores differences in views expressed by the children themselves, their parents, and HCPs. The research aims to answer the following question: what are the most important ways of participating in health care from the child’s perspective and the child perspective?

## Methods

### Design

The design uses the mixed methods approach of Q methodology. This methodology was developed to examine similarities and differences in participants’ views on a specific issue [20,21]. Participants rank statement cards that present different viewpoints on a given issue. Each individual ranking is then compared with the rankings of the other participants using factor analysis. Participants with common views are identified for the same factor [20]. Q methodology studies commonly use approximately 40 statements, but the number of statements can vary from less than 20 to more than 200 in studies with adult participants [22-24]. It is more important to cover all possible opinions on the topic in the statements than to have a fixed number of statements [23].

The Q set for this study, consisting of 25 statements for ranking, was formed based on the results of a literature review on children’s participation in health care situations [10]. Initially, 95 statements were formed from the themes of the literature review in English. Some of the statements had similar content and needed to be simplified to make them more accessible to 3- to 5-year-olds. The statements were discussed among all the authors, and duplicate or overlapping statements were removed. Once all the themes from the literature review had been incorporated into the statements, the authors agreed on a set of 25 statements. These statements were the same for all participants but were adapted in their formulation and pictorial representation to reflect the 3 different perspectives from which they would respond: child, parent, and HCP (Table 1). The statements were translated into Swedish by the authors. One of the authors speaks Swedish as a first language, while another speaks English as a first language. The translations of the questions, Q statements, and, later in the data analysis phase, the transcripts were checked and verified by all the authors, who are fully fluent in both languages.

**Table 1.** Q statements.

Number	Statements for children	Statements for parents	Statements for health care professionals
1	I want the doctor and nurse to talk to me too	My child wants the doctors and nurses to talk directly to her or him	Children want me to talk directly to them
2	I want the doctor or nurse to use words which I can understand	Doctor or nurse should use words that my child will understand	I should use words the child understands
3	I want to answer questions from doctor or nurse	My child wants to answer questions from the doctor or nurse	Children want to answer questions from me
4	I want to ask questions to the doctor or nurse	My child wants to ask questions from doctor or nurse	Children want to ask me questions
5	I can give my answers by pointing or nodding	My child can use nonverbal communication instead of talking to the doctor or nurse	Children can use nonverbal communication during the visit
6	I want parents and doctor or nurse to make decisions about me	My child wants me to make decisions with doctors and nurses	Children want the parent and me to make decisions during the visit
7	I want to make decisions except for difficult issues	My child wants to make the decisions, except for the difficult issues	Children want to make decisions, except for the difficult issues
8	I have important things to say before decisions are made about me	My child has important things to say before doctor or nurse and I decide on something about her or him.	Children have important information to share before the parent and I make decisions about them
9	As I grow older, I can make decisions on things concerning me	When my child grows older, he or she can decide on matters concerning her or him	When children grow older, they can decide on matters concerning them
10	I should be asked when decisions are made about me	My child should be asked before decisions are made about her or him	Children should be asked before decisions are made about them
11	I want to decide what to do when waiting for the doctor or nurse	My child wants to decide what he or she wants to do during the waiting time for the doctor or nurse	Children want to decide what they want to do during the waiting time to doctor or nurse
12	When I have answered many questions, I want to play	My child stops answering questions when he or she feels he has participated enough	Children stop answering questions when they feel they have participated enough.
13	I will resist things I do not want to do	My child will protest when he or she does not want to participate anymore	Children protest against procedures when they do not want to participate anymore
14	I want to do something else when the doctor or nurse speaks	My child wants to concentrate on something else during the health care visit when I talk with the doctor or nurse	Children want to focus on something else during the health care visit when I discuss with the parent
15	I want to quietly listen to doctor or nurse	My child wants to listen to the doctor or nurse but wants to stay quiet during the visit	Children want to listen to me but stay quiet during the health care visit
16	I want to talk to the doctor or nurse only when my parent is present	My child wants to talk to the doctor or nurse only when I am present	Children talk to me only when the parent is present
17	I want the doctor or nurse to listen to me too	My child wants the doctor or nurse to listen to her or him also	Children want me to listen to them also
18	I want my parents to let me talk like I want with the doctor or nurse	My child wants me to let her or him speak freely with doctor or nurse	Children want to speak freely with me, without the parent interrupting
19	I feel good when I make decisions together with my parents and doctor or nurse	My child feels that she or he is participating greatly when she or he decides something together with me and doctor or nurse	Children feel that they are participating greatly when they decide something together with their parents and me
20	I want the doctor or nurse to speak to me in a nice way	My child wants the health care staff to talk to her or him in a nice way	Children want the staff to talk to them in a nice way
21	I want to have choices from which to choose from during my health care visit	My child wants to have choices to choose from during the health care visit	Children want to have choices to choose from during the health care visit
22	I want to think before answering questions	My child needs time before answering questions from doctor or nurse	Children want to have time to think before answering questions
23	I want to make decisions together with the adults (my parents and doctor or nurse)	My child wants to make decisions together with me and doctor or nurse	Children want to make decisions together with their parents and me
24	I like to meet the same nurse or doctor every time I come to the health care center	My child wants to meet the same staff every time we come for a visit	Children want to meet the same staff members at every health care visit
25	I want the doctor or nurse to ask me before they are going to do something to me	My child wants doctor or nurse to ask her or him before they do any procedures with her or him	Children want me to ask them before doing any procedures

**Figure 1.**
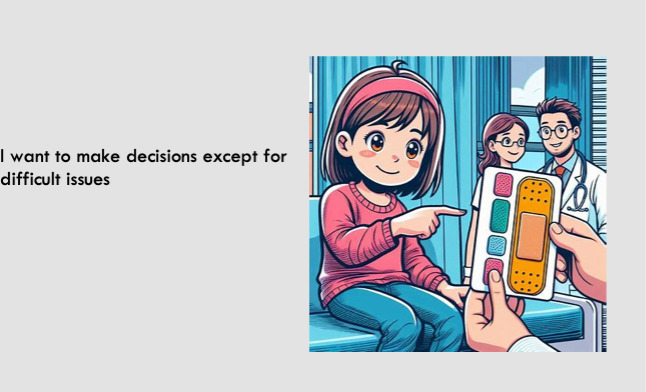
Q statement card for children.

Parents and HCPs were asked to respond on behalf of their child or patient, indicating their preferences. To illustrate the meaning of the statements, Microsoft Bing Copilot (version 2024 May) was used to draw pictures for the children’s cards (Figure 1). The content of the images and the desired meaning were discussed jointly by the authors during the development of the images. The first author also conducted a preliminary test with a convenience sample of 3 children of the appropriate age for the study to ascertain the images conveyed the intended meaning. The final selection was then made and agreed upon by all authors.

**Figure 2.**
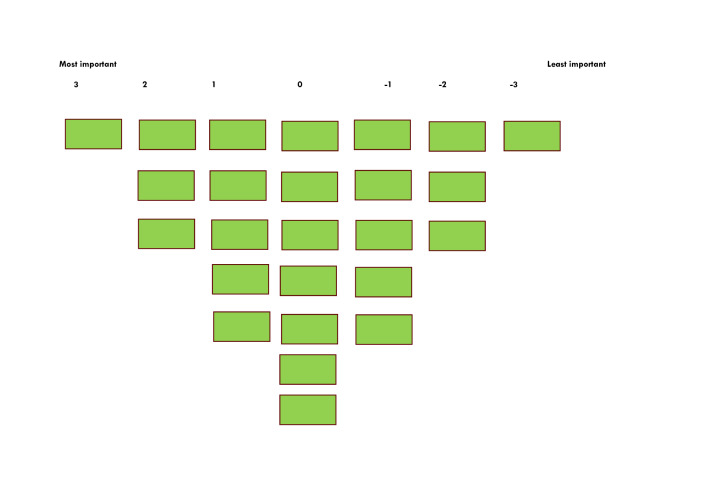
Q grid.

The Q grid, a fixed pattern in the form of a normal distribution for ranking, was chosen to range from least important (−3) to most important (+3), with the extremes of the pattern allowing only 1 statement (Figure 2).

After the participants had ranked the statements, postsorting interviews were conducted for all participants (Textbox 1). The postsorting interviews were conducted to let the participants provide rationale behind the Q sorting and to express their feelings and opinions while conducting the Q sorting [20,25].

Textbox 1. **Postsorting interview guide.**

Why did you choose to rank the statements in this order?

Why do you think that statement X is more important than statement Y?

Why do you think that statement X is the most or least important?

Are there other ways of involving children that are not mentioned in the statements?

### Setting

This study focuses on Child Welfare Centers in Stockholm Region, Sweden. These centers form a part of the national CHC program, which provides free, voluntary health services for children aged 0 to 6 years. The program is widely used and supports child development, parenting, and health promotion [26]. CHC nurses conduct regular health check-ups, including growth monitoring, vision, and balance tests. They also use activities such as drawing and memory games to assess motor skills, communication, and social interaction [27,28]. The goal is to support families and identify the need for further interventions for children [27].

### Participants

The participants were recruited via child welfare centers. Three child welfare centers in the Stockholm region participated in the study. One of the centers took part solely through staff interviews in order to obtain the required number of staff members for the study. Nurses from the other 2 centers recruited the child and parent participants through purposive sampling. Participants had to be a child aged between 3 and 5 years, or a parent of a child aged between 3 and 5 years, or a nurse or doctor working with children in this age group in a child welfare center, and they had to be able to understand and speak Swedish. Information about the study was sent to potential participants (children and their parents) prior to their appointment. This included a simplified version with pictures for the child. If a parent agreed to participate in the study, or to allow their child to participate, the nurses contacted the first author to arrange an appointment. Some parents were also asked during the appointment if they wanted to participate in the study, as the nurses and the first author agreed on days when the first author would wait for potential participants at the child welfare center.

The required number of participants was 12 or more in each category. There were no limits on how many participants of a certain age or with a certain amount of work experience needed to be recruited. Seventeen children agreed to be interviewed. However, only 12 of them could complete the sorting task and the subsequent interview. They did not complete the sorting for various reasons: they stopped answering questions, started playing instead of continuing the sorting, or could not concentrate on the questions and directed their attention elsewhere. None of the 3-year-olds (3 persons) who agreed to be interviewed could complete the sorting task. Fourteen parents agreed to be interviewed, but 1 decided not to complete the postsorting interview. Twelve HCPs agreed to be interviewed and completed the sorting and postsorting interviews. This resulted in a convenience sample of 38 participants who were willing to take part.

### Data Collection

If a parent agreed to participate in the study, the first author was introduced to them and their child by nurses. Statement ranking and postsorting interviews were conducted immediately after the child’s appointment, in a waiting or appointment room at the child welfare center and away from other patients or visitors. Nurses and doctors also conducted the ranking and interview at the child welfare center when their schedules permitted.

All participants were asked to provide the following background information: the age and gender of the child, the age group and gender of the parent, and the work experience and gender of the staff member. Children and parents were also coded together to enable a comparison between how they sorted items.

The child and parent participants completed the Q sorting exercise simultaneously, sitting side by side. The first author verbally explained the sorting procedure to the participants and also provided them with written information about the study. The first author then read and showed the Q statement cards with pictures to the child, while the parent sorted their statements independently. All participants received the same Q statements but written from the different perspectives of the participants (Table 1). After sorting, the final Q grid with the statement cards was photographed for analysis. Participants were interviewed about their motivations for sorting. These postsorting interviews were audio-recorded for the adult participants. The entire ranking situation, including the sorting and postsorting interview, was audio-recorded for pediatric participants. These interviews were transcribed verbatim in Swedish and, during the analysis, translated into English by the authors.

### Ethical Considerations

This study does not fall under the Swedish Ethics Review Act since the study does not collect sensitive data and is nonexperimental. However, the Swedish Ethical Review Authority has given an advisory opinion that there are no ethical objections to this study (Dnr 2024-02308-01). The study follows the ethical principles outlined in the Declaration of Helsinki [29]. Participation in the study was voluntary. Adult participants signed a consent form for their participation and an additional form if their child was participating. The children were verbally asked for their assent and were informed that they could withdraw from the study at any time. This study concerns children’s preferences and does not collect any identifying information. Any such information provided by participants during postsorting interviews was omitted or anonymized during transcription. Participants were given a cinema ticket as a thank you for taking part.

### Analysis

The factor analysis was carried out using the PQMethod software [30], a statistical program. First, data from the ranking of statements were entered into the PQMethod software. Centroid factor analysis, principal component factor analysis, and varimax rotation of factors were performed for each individual sorting. Then, based on the factors obtained, all participants were compared with each other. The common views on each factor were identified by distinguishing the preferred participation methods that the participants in the factor felt most strongly about. These extremes of +3, +2, −2, and −3 were then analyzed to characterize the common views of the factor. The participants associated with the factor were then identified. Several assessments were performed with a variable number of factors, and the optimal balance for most participants was identified when the number of factors was 3. This gave a good balance of most participants associated with the factors. The resulting factors included most adult participants, but only 6 out of the 12 children were identified to be associated with the factors. Therefore, the children’s Q sorts were also analyzed separately using the same factor analysis method and analysis of common views characterizing the different factors. Finally, the participants’ motivations for sorting from the postsorting interviews were used to corroborate and clarify their preferences for child participation.

## Results

### Participant Characteristics and Analysis Overview

The sample included 38 participants: 12 children, 14 parents, and 12 HCPs (see Table 2 for participant characteristics). The results of comparing the Q sorts of children, parents, and HCPs are presented as 3 factors representing the children’s different preferences for participation, followed by 3 factors from by-person factor analysis of the children’s Q sorts only. The Q sort values by factor are presented in Table 3. Finally, the alternative ways of participating, as suggested by the participants during the postsorting interview, are presented.

**Table 2.** Participant characteristics.

Participant group and characteristics	Factor all^a^ 1	Factor all 2	Factor all 3	Factor children^b^ 1	Factor children 2	Factor children 3
Children						
Age (y)						
5	1	1	1	2	2	1
4	0	2	1	1	2	2
3	0	0	0	0	0	0
Gender						
Boys	1	1	1	2	2	1
Girls	0	2	1	1	2	2
Parents						
Age range (y)						
26‐35	4	0	2	N/A^c^	N/A	N/A
36‐45	3	1	0	N/A	N/A	N/A
46‐55	1	0	0	N/A	N/A	N/A
Age not given	1	0	0	N/A	N/A	N/A
Gender						
Male	3	0	1	N/A	N/A	N/A
Female	6	1	1	N/A	N/A	N/A
Health care professionals						
Work experience with children (y)						
<2	1	0	0	N/A	N/A	N/A
2‐5	2	1	0	N/A	N/A	N/A
5‐10	1	0	1	N/A	N/A	N/A
10‐15	0	1	0	N/A	N/A	N/A
>15	1	3	1	N/A	N/A	N/A
Gender						
Female	5	5	2	N/A	N/A	N/A
Male	0	0	0	N/A	N/A	N/A

a ”Factor all” refers to the factor analysis of all participants' rankings.

b ”Factor children” refers to the factor analysis of children's rankings only.

c Not applicable.

**Table 3.** Factor Q sort values for each statement.

Number	Statement	Factor all^a^ 1	Factor all 2	Factor all 3	Factor children^b^ 1	Factor children 2	Factor children 3
1	I want the doctor and nurse to talk to me too	3	0	−2	−1	−1	−2
2	I want the doctor/nurse to use words which I can understand	2	2	0	−1	0	0
3	I want to answer questions from doctor/nurse	0	0	−2	2	1	0
4	I want to ask questions from doctor/nurse	1	−2	0	0	−1	−1
5	I can answer by pointing or nodding	0	1	2	0	0	−3
6	I want parents and doctor/nurse to take the decisions over me	−1	0	0	1	2	0
7	I want to make decisions except for difficult issues	−2	1	−1	1	2	0
8	I have important things to say before decisions are taken about me	0	1	3	0	0	0
9	As I grow older I can take decisions on things concerning me	1	−1	1	−3	1	0
10	I should be asked when decisions are made about me	0	−1	0	2	−2	0
11	I want to decide what to do when waiting for the doctor/nurse	−1	−1	−1	−1	0	1
12	When I have answered many questions I want to play	−1	−1	−1	2	1	0
13	I will resist things I do not want to do	1	0	2	0	−3	−2
14	I want to do something else when the doctor/nurse speaks	−2	0	−3	1	1	−2
15	I want to quietly listen to doctor/nurse	−3	−2	0	0	−2	0
16	I want to talk to the doctor/nurse only when my parent is present	−2	−3	−1	1	−1	0
17	I want the doctor/nurse to listen to me too	2	1	0	−1	2	−1
18	I want my parents to let me talk like I want with the doctor/nurse	−1	−2	0	0	−1	−1
19	I feel good when I make decisions together with my parents and doctor/nurse	0	3	0	1	0	2
20	I want the doctor/nurse to speak to me in a nice way	2	2	0	−2	0	0
21	I want to have choices from which to choose from during my health care visit	0	0	−2	0	3	−1
22	I want to think before answering questions	0	−1	0	−1	1	−3
23	I want to make decisions together with my parents and doctor/nurse	−1	2	2	−2	0	3
24	I like to meet the same nurse or doctor every time I come to the child welfare center	1	1	0	−2	−1	2
25	I want the doctor or nurse to ask me before they are going to do something to me	1	0	2	3	-2	1

a “Factor all” refers to the factor analysis of all participants' rankings.

b “Factor children” refers to the factor analysis of children's rankings only.

This study shows that children and adults have qualitatively different views, as only 1 of the child participants ranked the statements in a similar way to the parent. The perspectives identified in the factor analysis of all the participants’ rankings reveal 3 different approaches: involving children in communication during the health care visit, sharing decision-making with children, and, lastly, allowing children to set their own limits for their participation. Adult participants placed particular emphasis on HCPs’ communication with children and the establishment of a trusting relationship with them in the postsorting interviews.

### Factor All 1: Direct Communication Between the Child and HCPs

Nine parents, 5 HCPs, and 1 child associated with this factor. They felt strongly about HCP communication with children, with the highest-ranked statements concerning how HCPs interact with children as patients and provide children with the possibility to participate in discussions. One parent saw health care visits as a learning opportunity for children to learn to have a conversation.

*You should still teach the child that first the doctor has to say something and then they can say it. Not just throw out a bunch of words, but you should still try to learn to have a conversation.*

[Parent 4]

Communication was considered important for building a trustworthy relationship and ensuring a positive and comfortable health care encounter for the child. A positive and respectful way of communicating with the children was seen as the basis and an essential condition for the visit, more important than the children taking part in decision-making. Meeting the same staff was not necessary, but the communication skills of the HCP were highlighted for “inviting the child to participate in the visit.” Nonverbal communication was not ranked high in importance but was acknowledged during the interviews as an important means of communication for individual children. One HCP described this interpretation of signals as an important part of her communication skills.

*…it’s rather important that I as a healthcare professional can listen to them and see this and interpret their signals, do what they want and don’t want, with their body language instead.*

[HCP 9]

HCPs felt that most children wanted to participate in the visit. Similarly, parents belonging to this factor described their children as willing to talk and ask questions. Therefore, staying quiet and listening or using nonverbal communication (statements 15 and 5) were not important.

*She is not so interested in being quiet [laughs]*

[Parent 14]

Statements concerning decision-making and choosing from choices were mainly understood to refer to major decisions on treatment options and considered less important. Both parents and HCPs felt that children did not want such responsibility and doubted children’s understanding of the consequences of decisions. Some decisions, for example, diet changes for obese children, were not seen as suitable for children. While 1 parent felt that their child with more care needs and experience would need to be more involved in decision-making, other parents and HCPs stated that children were more interested in knowing what would happen during the visit than in making decisions with adults.

*And this idea that we should be able to make decisions during the care visit, I don’t think they care about that in the slightest, they just want to know what’s going to happen.*

[HCP 12]

Parents and HCPs felt that decisions should be age-appropriate and aligned with children’s cognitive levels. Allowing children to choose the order of procedures during a health assessment was considered valuable and within the limits of children’s decision-making abilities.

*… here he got to choose which arm you want the injection to be taken. It’s the child’s body and I think it’s important for the child to say that this arm will feel better to me. You might have pain in some place before.*

[Parent 4]

Sometimes procedures had to be done regardless of choice, making mutual understanding between the child and HCP essential. Protesting was seen as a way for children to express emotions, and while protests were respected and could delay care, they did not always change the outcome.

### Factor All 2: Understanding and Shared Decision-Making

Three children, 1 parent, and 5 HCPs were associated with this factor. They felt strongly about participating in decision-making and thought it was important for children to understand and follow the events of the health assessment at the child health care center. They also did not think that the parents’ presence was necessary for children to talk to the nurse or doctor. HCPs felt they needed to quickly build a relationship with the child and show that they were friendly and not scary. Using translators in the situation hindered this relationship-building, but regular contact with the same HCP helped. Another effective approach was to prepare the children at home before the visit. Like in Factor all 1, a positive and comfortable health care encounter with direct communication with children was considered the foundation for a visit, but this factor emphasized shared decision-making with parents and HCPs. This required understanding of the discussion during the visit.

*Sometimes certain words become difficult, and she can't quite keep up.… But this thing about making decisions together. It’s linked to the fact that if you understand the meaning of it and in the same way, it’s important to do it together.*

[Parent 12]

Shared decision-making with parents and HCPs was found to be valuable, at the appropriate level of decision-making for children. Even though children might not fully understand what it involved until it was imminent, some decisions were beyond children’s capability. Participants associated with this factor felt that children could decide more as they grew older. On the other hand, relevant decision-making for children was seen as children deciding over a range of options presented, giving them a sense of control over the situation.

*They can choose the color of the paper they draw on, for example, for their drawing. And they can choose the order in which we do things, whether we weigh them first or check their eyesight first. But I don’t think the difficult decisions feel important for the child to choose. Instead, they think it’s nice that someone else decides. For example, whether to take a vaccine. They can’t decide that.*

[HCP 6]

### Factor All 3: Responsive and Child-Led Participation

Two children, 2 parents, and 2 staff members were associated with this factor. Their preferences are interpreted as the children setting limits on their participation. These limits include consulting them before procedures, sharing decision-making, the possibility of protesting, and the use of nonverbal communication. Asking the children before procedures (statement 25) was motivated by the aim of involving them when possible, allowing them to make decisions based on their age and situation. Participants stated that children would participate for as long as they were interested, after which they would move on to playing. One parent noted that varying levels of participation meant that offering choices during health assessments was less important.

*I thought it was like this, yes, sometimes it is important. Sometimes it’s a bit varied from day to day. Sometimes it’s really important to get him to speak up and tell me about something and ask me something. Sometimes let me sit in a corner.*

[Parent 9]

When children grew older, they could make more decisions, but at this age, some decisions on treatments were too complex for children to make. Children’s participation was vital for some procedures; therefore, a gentle approach was used, with the possibility of postponing the procedure.

*…if you take something as concrete as checking vision. You almost have to try somehow to make the child want to do it, because otherwise you might miss vision problems and there will be problems later in school or something, or headaches...*

[HCP 7]

HCPs found it important to talk directly to children, even though they felt that children rarely asked questions of them. Similarly, a parent thought that the questions should be directed at children. If the child was unwilling to answer or colored the answer with fantasy, the parent would step in.

*Yes, but if he wants to answer, he can. And then... we had a visit to the BUM [medical center] and task: does he eat vegetables? Yes, ask him. And then he unraveled the whole vegetable stash as well.*

[Parent 9]

The importance of nonverbal communication was recognized. A parent explained that their child was very shy but willing to participate in their own way.

*…it’s very much that if she can respond with body language instead, or she can be quiet if she wants to, and a lot of things like that, as she wants it. She doesn’t usually, and neither do her siblings, who want so much... care whether decisions are made with them or not.*

[Parent 6]

### Factor Children 1: Included in and Setting Their Own Terms for Participation

Three children were associated with this factor. They felt strongly about receiving information, being consulted prior to procedures, participating in discussions, and choosing how long to participate. This gave them a sense of control within the health care situation. As with Factor all 3, the children set their own terms for participation but placed the least importance on making decisions alone or with parents and HCPs. In the following situation, the child suddenly starts playing while still answering questions from the interviewer. She understands the question in a practical way. Furthermore, it can be interpreted that decisions regarding play are important to her. Her actions demonstrate how she sets the terms for her participation, showing agency.

***Interviewer:** Okay. [child walks away from the table where the interview is being done] Are you going on the slide? Was it hard to make the cards? You turn your head. Was it easy? Mmm. Is there anything that you would like to do when you are with the doctor or nurse that was not included? Is there anything that you would like to do when you are with the doctor or nurse? Apart from going down the slide. Is there anything else you would like to do?*

***Child:** Swing.*

***Interviewer:** Sing?*

***Child:** Swing.*

***Interviewer:** While you are waiting? Or after you have been to the doctor or nurse?*

***Child:** Mmm.*

***Interviewer:** So, is it important what you do while you are waiting?*

***Child:** Mmm.*

[Child 3]

This example presents a comfortable level of decision-making for the child. The statement 7 (“I want to decide on easy things but not difficult”) illustrated with a child picking a Band-Aid reflects a familiar, practical choice.

***Interviewer:** We’ll take this one which is important, first that pile. I put them on the floor. If you look at them and say what is the most important of all these. Then it’s just one card. See, [child’s name]? Should I read them all? This one? [reads aloud the card the child is pointing to] I want to decide on easy things but not difficult ones. Is it most important when you come to the nurse?*

***Child:** [inaudible] band-aid.*

***Interviewer:** Yes, to be able to choose band-aids?*

***Child:** Yes.*

[Child 9]

### Factor Children 2: Small Choices, Meaningful Outcomes

Four children were associated with this factor. They felt strongly about having a choice of options (statement 21) and making decisions about minor issues, while leaving major decisions to parents and HCPs. These choices could be understood as play-related, perceiving the visit as a meeting with HCPs and playtime, and choosing what to play with while waiting as meaningful decisions. Although nurses also use games and play as a method of observing children’s development during health assessments, this may explain the child’s confusion.

***Interviewer:** Is it good like this? Is there anything else you would like to do when you come to meet the nurse?*

***Child:** Read a book.*

***Interviewer:** Read a book, okay.*

***Child:** Playing with dolls.*

***Parent:** You want to be in the waiting room.*

***Interviewer:** There are lots of things you want to play with. Mmm. Okay.*

***Child:** Doll. Slide. Read a book.*

***Interviewer:** So, before the visit or after the visit?*

***Child:** After the visit.*

[Child 17]

The children actively ranked their preferences during the interview but struggled to motivate their choices. One child asked for help from their mother, who then interpreted the child’s response.

***Interviewer:** Why did you choose to sort the statements like this? Was it a...?*

***Child:** Mum.*

***Interviewer:** Wasn't it you who decided?*

***Child:** No.*

***Interviewer:** No?*

***Parent:** It was you who decided all by yourself.*

***Child:** I don't know.*

***Parent:** You don't know why you chose that?*

***Child:** No.*

[Child 1]

One parent noted that the child chose opposite preferences, likely due to interpreting the statements differently.

*…this one sounds like he’s going to choose different things when he leaves, but for me it’s different choices to choose whether he wants to... start by weighing himself or measuring himself first…*

[Parent 1]

In a few cases, participating in the process was more important than the result. One child took over the following postsorting interview and proudly explained her ranking but left out the details of her priorities.

***Child:** Now you got to make piles.*

***Parent:** I got to make piles too. It is the same as you. What I think is important [talking to the child]*

***Child:** What is important?*

***Parent:** That doctors and nurses listen to... to the child? I don't know what you chose.*

***Child:** I chose all the things that were not important and all the things that were important and in between.*

[Parent 4 interview]

Some children associated with this factor were described by parents as talkative and curious, which matched their ranking. They ranked the statements about being quiet, being asked before procedures or decisions affecting them were made, and the possibility of protesting the lowest. These children seem accustomed to expressing their opinions and are unaware of situations in which they cannot do so verbally.

### Factor Children 3: Trust Through Familiarity and Shared Decision-Making

Three children were associated with this factor. They prioritized decision-making with parents and HCPs as well as a positive and comfortable health care encounter by HCPs, similar to Factor all 2. However, unlike Factor all 2, participants in this factor considered it important to meet the same HCPs (statement 24) and did not consider it important for HCPs to talk to children as well (statement 1). This suggests the need for parents to be present and supportive during meetings with HCPs, and for children to be willing to build relationships with HCPs. In the following example, the child’s wish to repeat the vaccination suggests an optimal level of shared decision-making and trust with the HCP.

***Interviewer:** Is there anything else you would like to do when you come to the nurse that is not on these cards? Is there anything you absolutely want to do when you come to the nurse?*

***Child:** Hmmm.*

***Interviewer:** That might be a difficult question because we have so many cards here.*

***Parent:** Exactly. Is there anything [child’s name]? Is there anything you would like to do?*

***Child:** No.*

***Interviewer:** So, then you're done with it.*

***Child:** Syringe.*

***Interviewer:** What did you say?*

***Child:** Syringe.*

***Parent:** Would you like a shot [vaccine, author’s remark]? You've had a shot.*

***Child:** Yes.*

***Sibling:** He really likes syringes now.*

***Interviewer:** Oh, so it went so well that you thought I could do that next time too. That’s great. Okay. I think I’m done too. Then, I thank you for the interview.*

[Child 16]

Nonverbal communication was ranked as the least important. One child who relied on nonverbal communication during the interviews ranked it low and prioritized decision-making instead. This suggests that the feeling of being involved mattered more than the way of communication, as confirmed by her parent:

*She just wants to get... So, in her own way, be quiet, respond with thumbs up, thumbs down. Then she’s usually happy.*

[Parent 6]

### Additional Suggested Ways of Participating

In the postsorting interview, participants were asked for other ways children could participate in health care situations. Children mentioned different toys for the waiting room, while adults recommended postponing procedures to a later time if the child was not ready. They highlighted the importance of preparatory information, which helped both children and parents make visits run smoothly. Using pictures to aid communication and allowing children to play with a toy before talking to HCPs were also found to be effective strategies. Reflecting on the visit afterward to assess the child’s experience was suggested. The value of the HCP’s skills and parental involvement in the process was emphasized, as praise or confirmation from the parent while the child is answering questions promoted their participation, whereas a lack of parental engagement in the situation demotivated children.

*Or being a parent and looking at the clock all the time and just being stressed and going to work. That doesn’t work either. Most often, many times it doesn’t work. You have to come back again, so that the child realizes that we don’t have time to be here.*

[HCP 8]

HCPs also felt that they needed to be flexible and provide options for children. Despite the many obligatory examinations and decisions that children could not influence, HCPs recognized that there were always ways to involve them in the visit

*I think that in everything we do with children there is always a choice. They can always be allowed to choose which band-aid, which arm.*

[HCP 12]

## Discussion

### Principal Findings

The aim of this study was to understand children’s preferences for participation in health care situations and to examine them from the child’s perspective as well as from the child perspective. This study sheds new light on the preferences of preschool-aged children. To our knowledge, this is the first study to compare the perspectives of preschool-aged children, their parents, and health professionals on children’s participation. The key findings can be summarized as follows.

First, preschool-aged children are happy to leave major decisions to adults, but they want to be in control of health care situations. Similarly, Darcy et al [31] described how children undergoing cancer treatment wanted to control how, when, and at what pace procedures were conducted, and how much they participated in them. According to Moore and Kirk [32], this type of decision, which does not alter the outcome, is considered tokenistic because the planned procedures will be carried out regardless. The current study found that this kind of control is important for preschool-aged children, giving them an opportunity to participate and set their own limits to participation. This relates to the findings by Carlsson et al [33] on how HCPs considered participation as a prerequisite for care. They tried to “make the most of the moment” by involving children at every step, for example, by letting the child choose if they wanted to sit or lie down during the examination. Decosta et al [34] found that actively involving children in the preparation of medical procedures made diabetes education more meaningful, as it engaged them in their own care in an age-appropriate way. Furthermore, positive feedback, play-based approaches, and playful communication encouraged the young (<7) children’s participation. These approaches were designed to help children gradually understand and engage more with their care. They are valuable for encouraging children to participate, as the present research supports the idea that some medical examinations depend on children’s participation. For example, a vision check requires the child’s participation. Children’s protests or nonparticipation could postpone the examination to later and result in unnecessary suffering of undetected and treatable vision problems.

Second, a supportive and present parent appears to be important to children. Harlow et al [35] define parental presence in pediatric hospital care as being physically or emotionally present with their child, reducing the child’s anxiety and providing emotional support. Besides the emotional support, children seek their parents for advocacy and sense of security during hospitalization [36,37]. Another aspect is learning how to participate with the help of supportive and encouraging adults. In turn, adults need to strike a balance between protecting and encouraging participation [19], or between intervening and giving autonomy [38], in order to provide children with an opportunity to express their views [16]. This means focusing on the child at the center of the care, as outlined in the CCC principles, and giving the child a voice [1,2,16].

Third, participants in the study suggested other ways of involving children, such as providing preparatory information with visual aids or postponing the procedure to a later date. Previous studies have demonstrated the effectiveness of visual aids and digital tools in preparing children for procedures and facilitating their participation in daily discussions about their care in hospital wards [27,39,40]. Digital tools could standardize the information provided to children. A digital application could facilitate children’s participation by providing an understanding of what will happen during the procedure [41], while enabling HCPs to identify areas where the child requires further information before the procedure begins. Another suggestion from the participants was to ask children about their experience of the health care situation. This idea is worth exploring further in future research. Asking children at the end of a visit about their experience would provide valuable insight and help HCPs prepare for subsequent encounters with children.

Finally, children and their parents perceive and interpret the world differently. While preschool-aged children are still developing their language skills and abstract thinking abilities [42], they are capable of processing the information presented to them [43] and actively shaping their social environment as competent participants [13,14]. The aim of this Q methodology study was to identify and describe the common perspectives of the participants regarding child participation in health care situations. The results comparing the 3 different views (children, parents, and HCPs) and motivations for the rankings suggest that the resulting perspectives are coherent. Children may not be able to justify their choices, but this should not diminish the importance of their opinions, nor should it be a reason to override them if they differ from the views of adults [16]. Children’s preferences identified in this study are in line with the core principles of CCC: trust, respect, autonomy, self-determination, and decision-making regarding their care together with parents and HCPs [2]. The current study showed that parent proxy does not always provide a correct picture of the phenomenon. Therefore, the perspectives of children should be sought in research and health care practice, as well as in other disciplines, and practitioners should be made aware of this perspective, as set out in the UNCRC [9]. This document should be understood as legally binding within state parties [9], ensuring that children are able to express their views. As the children were unable to justify their rankings, it would be interesting to explore this further.

### Strengths and Limitations

This study has some limitations. It revealed various perspectives on children’s preferences regarding participation in health care situations. However, it does not indicate how prevalent these preferences are among preschool children. The Q statements were developed from themes identified in a literature review, simplified into age-appropriate Swedish, and supported by animations. To minimize potential misunderstandings, only participants who were fluent in Swedish were included in the interviews and analysis. Prior to the study, the meaning of the images provided with the statements for children was discussed with a convenience sample of children. Nevertheless, misunderstandings regarding the language may still have occurred. Conducting a pilot study could have been helpful. Children and their parents were recruited by nurses at Child Welfare Centers. This may have introduced a bias toward more outgoing children, which could have influenced the study’s outcomes. Appointments scheduled late in the afternoon, after a full day at preschool, may have reduced the children’s focus, particularly among the 3-year-olds, making it challenging for them to complete the statement ranking task. Conducting the ranking task alongside their child may have influenced some parents’ responses if they overheard the interviewer’s conversation with their child. Nevertheless, most parents were highly engaged in the ranking process, as they found the number of statements substantial and did not have time to listen to their child’s interview.

To our knowledge, no Q methodology studies have been conducted on children younger than 5 years. The number of statements varies greatly from less than 20 to more than 200 in Q-methodology studies [23,24]. In this study, coverage was achieved without redundancy, making additional statements unlikely to expand the viewpoint space meaningfully. Furthermore, Q sorting can be difficult, demanding, and time-consuming [20]. A more concise set reduces participant fatigue. The 25 statements in this study with child participants align with established practice while maintaining methodological rigor.

### Conclusion

In this study, 4- and 5-year-olds participated enthusiastically in the ranking process. This method also enabled us to compare the children’s perspectives with the efforts of parents and health professionals to understand them and, in some cases, their ability to explain the children’s rankings. None of the 3-year-old participants could complete the ranking procedure. It would be interesting to explore whether 3-year-olds share the perspectives identified in this study or whether they have different views, but different methods are needed for this.

## Abbreviations

CCC: child-centered care
CHC: child health care
HCP: health care professional
UNCRC: United Nations Convention on the Rights of the Child
